# Participants’ Perceptions of Advantages and Drawbacks of “Drop-In” Versus “Closed-Group” Formats Related to Cancer Bereavement Program Delivery

**DOI:** 10.3390/curroncol32090505

**Published:** 2025-09-10

**Authors:** Yoojung Kim, Carmen G. Loiselle

**Affiliations:** 1 Division of Experimental Medicine, Faculty of Medicine and Health Sciences, McGill University, Montreal, QC H4A 3J1, Canada; carmen.g.loiselle@mcgill.ca; 2 Gerald Bronfman Department of Oncology and Ingram School of Nursing, Faculty of Medicine and Health Sciences, McGill University, Montreal, QC H3A 2M7, Canada

**Keywords:** closed-group, open-group, program format, cancer, bereavement, support program, community-based, qualitative research, caregivers, family experiences

## Abstract

Having opportunities to readily access bereavement support for people affected by the death of a loved one is central to any comprehensive approach to cancer care. *Hope & Cope*, a community-based cancer support organization in Montreal, Quebec, Canada, offers professional- and volunteer-led bereavement programs in two formats: “drop-in” (open as needed) and “closed-group” (structured). This qualitative study explored contributions and potential drawbacks of these two-program delivery formats as reported by bereaved participants (*N* = 18). Semi-structured individual interviews were conducted according to groups: Drop-in (*n* = 7) and closed-group (*n* = 11). Audio-recorded interviews (lasting between 30 and 60 min) were transcribed verbatim. Data were analyzed using thematic analysis. Three themes were revealed: (1) Program structure according to grief timeline, (2) Flexibility in the choice of topics and impact on grief experiences, (3) Grief support dynamics in relation to group composition. Findings indicate that drop-in provided “as-needed” tailored support, whereas closed-groups ensured consistency in attendance. Some drawbacks included high attendance turnover in the drop-in and less relevant topics in the structured closed format. Supportive interventions should continue to be tailored to people’s profiles and preferences, not only for content but also for delivery formats.

## Simple Summary

The impact of cancer does not end at death for family members. Person-centered bereavement support is vital to ensure it meets the needs of affected individuals. The purpose of this qualitative study was to explore participants’ experiences while attending a “drop-in” (as needed) or a “closed-group” (consecutive sessions) community-based bereavement program. Through in-depth individual interview analysis, findings suggest that participants appreciated flexibility in attendance and ongoing access to support in the “drop-in” group, while the “closed-group” format favored relationship building among attendees in similar grief stages. Some drawbacks for “drop-in” included unpredictable attendees’ turnover, whereas the “closed-group” followed a structured way of addressing bereavement topics with less flexibility in meeting the changing needs of attendees. Insights gained can help optimize the delivery format and content of similar bereavement programs to best meet the needs of bereaved individuals.

## 1. Introduction

Losing a significant other to cancer is a distressing experience, particularly for family members who acted as primary caregivers throughout the illness trajectory [1]. Oftentimes, distress emerges at the time of diagnosis, causing a recalibration of family values, emotions, cognitions, and behaviors [2,3,4]. Unpredictability linked to cancer often negatively impacts families over time as they deal with feelings of uncertainty, guilt, inadequacy, and social isolation—both during the progression of the illness and following death [5,6,7,8,9].

Bereavement support in oncology plays a critical role in helping families after the loss of a loved one. Cancer care teams have been identified as key figures in shaping the bereavement experiences [10], with nurses noted to be uniquely positioned to recognize and address the needs of affected individuals [11,12]. However, family members frequently encounter a wide range of unmet needs during bereavement, [10,13,14] with 10 to 20% developing grief disorders [15,16]. To address access issues, community-based organizations are increasingly providing support to individuals following the death of a family member and bereavement [17,18]. As such, they are becoming integral partners in care within and external to clinical settings.

Bereavement interventions often include social, emotional, physical, and informational/instrumental support [17,19,20]. These are delivered in various formats including in-person, virtual, or hybrid as well as individual or groups facilitated by professionals, volunteers, and/or both [21,22,23,24]. Bereavement support groups, where people with shared experiences meet under the guidance of a facilitator, for instance, are commonly offered by community-based organizations [25]. Furthermore, support groups can be categorized as “drop-in” open-groups, where individuals may join at any time and attend for as many sessions as needed, or closed-group, where the same individuals begin attendance at the same time and are expected to attend a determined number of sessions [23,26].

*Hope & Cope* is a community-based organization located in Montreal, Quebec, Canada. To support bereaved individuals, *Hope & Cope* offers distinct bereavement programs including *Mourning Walk* (MW) and *Living with Loss* (LWL) [27]. *Mourning Walk* is an open-group/drop-in program offered in-person on a weekly basis, with each session lasting 1 h. Situated in nature (i.e., Mount-Royal park), the program is open to individuals at any stage of bereavement and offers a setting where they can share their experiences with the facilitated guidance of a trained volunteer [28]. *Living with Loss* is a closed-group program, consisting of 8 sessions delivered on a bi-weekly basis. It is open to individuals who lost a loved one to cancer within the last two years. Facilitated by a social worker, each of the sessions lasts 1.5 h with prior set objectives and grief-related topics such as feelings and emotions associated with grief, coping strategies, and finding acceptance (Supplementary Materials Figure S1). *Living with Loss* can be delivered both in-person and virtually [29].

Support group interventions have been shown to help bereaved individuals build social connections and normalize their grief experiences [30,31]. In addition, some programs have been effective in reducing distress and improving coping abilities [32,33]. Although the benefits of such programs are clearly documented, little is known about how divergent formats may impact participants’ experiences. The purpose of the present study is to deepen our understanding of how bereaved individuals perceive different support formats. More specifically, the study objective was to document participants’ perspectives after attending a drop-in (i.e., *Mourning Walk*) or a closed-group (i.e., *Living with Loss*) program.

## 2. Materials and Methods

### 2.1. Study Design

A descriptive qualitative design was used to gain deeper insights into participants’ experiences attending distinct bereavement programs. Open-ended interview questions were developed based on Sekhon and colleagues [34] framework related to the acceptability of healthcare interventions. Thematic analysis to identify emerging themes [35]. This study is reported in accordance with the Consolidated Criteria for Reporting Qualitative studies (COREQ) 32-item checklist (Supplementary Materials Figure S2) [36].

### 2.2. Participants, Setting, and Procedures

In this qualitative study, a convenience sampling approach [37] was used to recruit individuals who were 18 years of age and older, could communicate in English, provide informed consent, and had registered for the *Living with Loss* or *Mourning Walk* program offered by *Hope & Cope*. The study sample consisted of 18 individuals—11 *Living with Loss* registrants and 7 *Mourning Walk* registrants. All *Mourning Walk* and *Living with Loss* sessions were delivered in person. The *Living with Loss* program took place at the Hope & Cope Wellness Center, and the *Mourning Walk* program took place at Mount-Royal Park, both located in Montreal, QC, Canada.

All *Living with Loss* and *Mourning Walk* program registrants were first asked by their respective program coordinators if they gave permission to be contacted for research purposes. Upon their approval, a total of 24 program registrants (16 *Living with Loss* registrants and 8 *Mourning Walk* registrants) were contacted by the first author by phone to inform them about the study and its purpose: to better understand their experiences with bereavement programs in order to improve the programs and more effectively meet the needs of current and future users. Interested individuals were screened for eligibility and were sent an e-consent form by email. Once informed consent was obtained, each participant was assigned a unique, randomly generated ID code to ensure anonymity. All participants received $10 compensation for each study task (e-questionnaire and interview) at the end of their study participation.

### 2.3. Data Collection

**Figure 1:**
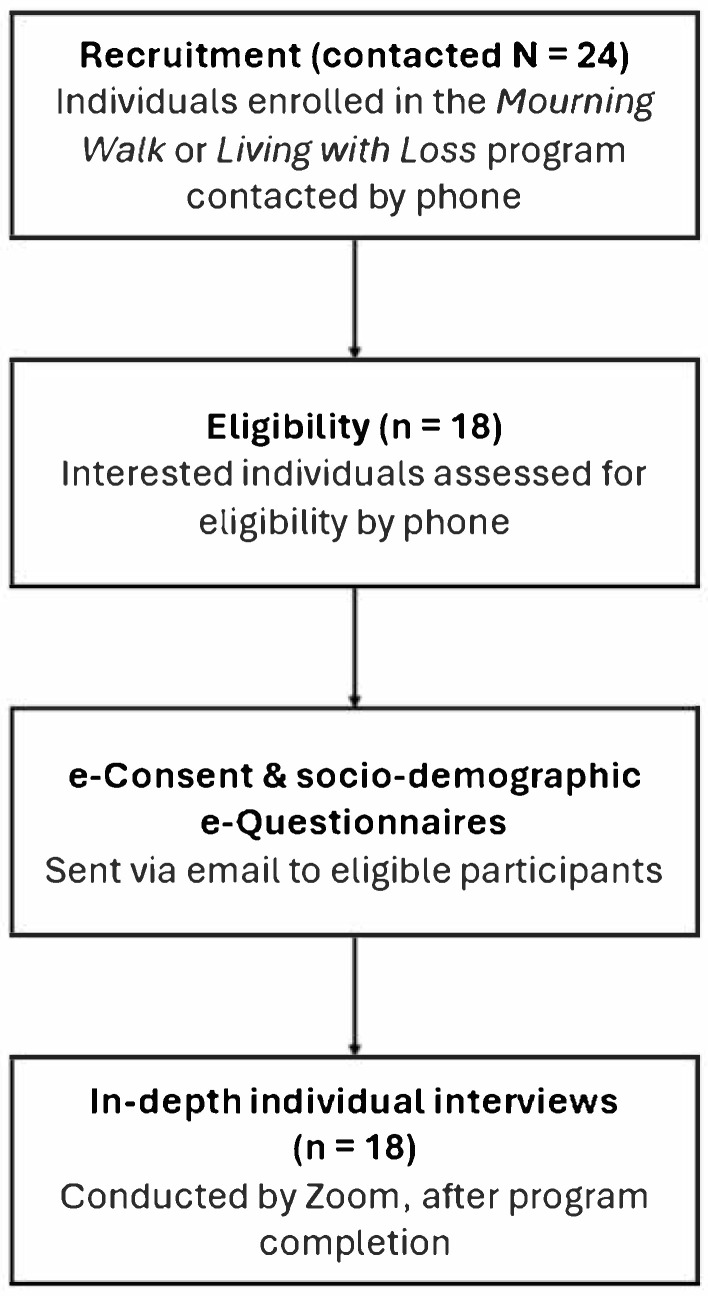
Study flowchart.

Figure 1 presents the flowchart for the data collection process. Data collection occurred between May and December 2024.

#### 2.3.1. Socio-Demographic e-Questionnaires

A socio-demographic sheet was sent electronically to participants and completed online using Qualtrics, a secure electronic data capture system. Descriptive statistics were computed to summarize participant characteristics using the IBM SPSS Statistics software (Statistical Package for Social Science), version 29 [38].

#### 2.3.2. Semi-Structured Interviews

Each participant was contacted to schedule their one-on-one interview. These were conducted at program completion (post 8-sessions for *Living with Loss* and within one year of attending *Mourning Walk*). Interviews were conducted by the first author using either Zoom’s video conferencing platform, in-person (at Jewish General Hospital, Montreal, QC, Canada), or by phone, based on participants’ preferences. Interviews were audio-recorded and lasted between 30 and 60 min. The interview guide (Table 1) consisted of seven open-ended questions using the Sekhon and colleagues’ guide on healthcare intervention acceptability [34]. This guide assessed components such as affective attitude, burden, perceived effectiveness, intervention coherence, self-efficacy, and ethicality. For example, to assess participants’ affective attitudes and perceived program effectiveness, we asked, “Is there anything you liked about (the program)?” and “To what extent do you think (the program) made a difference in the lives of people attending it?” respectively.

### 2.4. Data Analysis

#### Qualitative Data Analysis

Using an inductive approach, reflexive thematic analysis [35,39] was conducted manually using Word and Excel. The six-phase process developed by Braun and Clarke [35] was followed. In phase 1, interview content was transcribed verbatim. The data were thoroughly and independently read and re-read by the first author and a member of the research team, Saima Ahmed, Ph.D. (S.A.). Y.K. coded the transcripts in Word, highlighting specific sentences and labelling the phenomenon of interest with concise words or phrases (e.g., group dynamic, time restriction, personal needs) (phase 2). Initial codes were reviewed and validated by S.A. to ensure accuracy and reliability. Disagreements were discussed among the two coders until consensus was reached. In Excel, codes were refined and categorized into themes and subthemes until thematic saturation was achieved (phase 3). These were then reviewed and discussed with the senior author (C.G.L.) (phase 4). The latter involved assessing the essence and representativeness of each theme and subtheme. Y.K. and C.G.L. refined labels and definitions for each theme and subtheme to ensure optimal representation of the dataset and related research questions (phase 5). These themes and subthemes were further organized and reported, while being supported by relevant participant quotes (phase 6).

## 3. Results

### 3.1. Socio-Demographic Characteristics

Of the contacted 24 program registrants (for the two programs: *Mourning Walk* and *Living with Loss*), 75% agreed to take part in the study. The resulting sample (*N* = 18) included 16 women and 2 men between 31 and 89 years of age (Table 2). 83% (*n* = 15) were widowed and identified as Caucasian. All participants reported not currently living with someone and not having dependents. The sample was well-educated, with 60% (*n* = 11) having a university degree (either undergraduate or graduate). Most were retired (67%, *n* = 12), and 78% (*n* = 14) reported having lost a spouse.

### 3.2. Qualitative Results

Three main themes were identified from interview data: (1) Program structure according to grief timeline, (2) Flexibility in the choice of topics and impact on grief experiences, and (3) Grief support dynamics in relation to group composition. Each theme highlights advantages and potential challenges reported by participants who experienced either open or closed-group formats. Table 3 summarizes main themes and subthemes, ordered from abstract to more concrete concepts. These are organized according to grief timeline, topic programming, and group membership. Each theme and subtheme are reviewed in turn with relevant quotes from participants.

#### 3.2.1. Theme 1. Program Structure According to Grief Timeline

**Ongoing access to support.** All open-group participants in the Mourning Walk program appreciated the ongoing nature of the program, where they could attend for as long as they personally needed. Consistent access to support on a weekly basis provided comfort, as an open-group participant stated, “*I think just knowing, even if you don’t go every week, knowing that it’s there as a structure is actually very nice. I think it’s a kind of supportive thing in the background, knowing that if you had a bad week or, you know, had a trigger […] that you could go.*” (female, age 77, widowed) It also served as a source of support as they navigated the emotionally unpredictable and ongoing nature of the bereavement process. This was underscored by two participants:

“*The grief is the ups and downs, and when you’re having a down and then you have the walk, it was like ‘Oh, I’m really happy,’ you know? That is there. And then after that, you feel good, and then if you feel like going, you go if you can. And if you cannot, well, you just hope for the next time that you can go and share.*”

(female, age 59, widowed)

“*We’re always going to have the grief. He’s always going to be there in my heart. There’s still going to be memories that are always going to be there. His name still pops up in the family meetings, reunions or whatever. I still think of him.*”

(female, age 68, widowed)

The importance of consistent support was also highlighted by more than half of the closed-group (i.e., Living with Loss program) participants (7/11). One person emphasized that, “*grieving, first, is in no way linear. There’s not one person that grieves the same way,*” (female, age 64, widowed) and another stated, “*it’s something that you never stop going through and something you never forget,*” (male, age 31, lost parent) both acknowledging that everyone grieves at their own pace and grief can feel lasting. At the end of the program, many closed-group participants (7/11) expressed desire for program continuity, feeling they were no longer receiving the support they once had. A participant shared, “*It’s a little sad because it helps me when I’m down, that I know ‘Oh, I have a meeting, and I can share my feelings.’*” (female, age 65, widowed). Similarly, another commented, “*It just bothers me more as a group that we were going to walk out. Walk out of the front door and everybody scatters a different way, and it’s like, ‘OK, bye. Thanks. It’s nice to see you. Goodbye. Have a good life.’ I think we all maybe, I don’t say, we owe it to each other, but I think we can all still help each other.*” (male, age 77, widowed)

A few participants (4/11) questioned the length of the 8-session closed-group Living with Loss program: “*Is it long enough? Maybe not.*” (female, age 65, widowed). Several participants (5/11) reported feeling they could benefit from additional sessions, especially those who were not able to attend all sessions due to scheduling conflicts. With a limited number of sessions, they felt left behind and unable to fully benefit from the program, making their experience briefer than they would have liked. One participant noted, “*Because I missed a few meetings, I feel like I’m a bit disconnected now. It was short. It was too short.*” (female, age 62, lost parent) Overall, even though most closed-group participants wished for more sessions, they appreciated the opportunity to connect with other bereaved individuals and receive social and emotional support. One participant commented: “*Endings are hard, but you have to appreciate that is happening because it’s not happening anywhere else that I know of in public, in, you know, meeting people.*” (female, age 73, widowed)

**Perceived progress.** Most open-group participants (6/7) clearly described the progress they felt they had made because of taking part in the program. This included:

“*Talking with everybody, I realized the work I did through those years.*”

(female, age 59, widowed)

“*I think it [Mourning Walk] made an immense difference in my life. […] it was just sort of like the departure point for a journey that obviously has not ended, but where I can look back and see a long, very winding road.*”

(female, age unknown, lost parent)

Conversely, the closed-group, while some reported feeling different since their initial program attendance, others were uncertain as to whether they felt progress in their bereavement. For example, a participant commented, “*Supposedly we will get stronger. I don’t feel stronger for sure. I don’t feel stronger yet.*” (female, age 73, widowed)

**Consistent support.** Both open and closed-group participants emphasized the importance of regular, consistent sessions to maintain group dynamics and a sense of sustained support. Regular sessions provided on a weekly basis were appreciated by open-group participants: “*You know that apart from 2 weeks in the summer and the one week maybe at Christmas […] It’s there.*” (female, age 77, widowed) Thus, this seemed essential to the participants, offering reassurance and comfort in knowing they had support whenever they needed it.

The importance of having regular sessions was also emphasized by closed-group participants, especially after experiencing a break longer than the planned bi-weekly schedule. A participant stated, “*when there’s interruption of the [Jewish] High Holy days, it [the group] loses the essence. If it had been continuous, then I think it’s much better.*” (female, age 77, widowed) However, spacing between sessions also benefited some participants by providing time to process and apply what they had learned. A participant reported, “*I think just in terms of the way it was spaced out, I think it was good because I’m able to process things for a bit after each session. If it was all 8 weeks one after another, it might feel a little quick. So I think like it helps process things and then go through things.*” (male, age 31, lost parent)

#### 3.2.2. Theme 2. Flexibility in the Choice of Topics and Impact on Grief Experiences

**Balancing dynamic and timely needs.** As an open-group, Mourning Walk did not contain set objectives for each session. This allowed participants to feel at ease sharing whatever they needed at the moment. A participant noted, “*This [Mourning Walk] was appealing to me because it was very unstructured in that way. So it felt like people could talk about whatever aspect they wanted to in no particular order.*” (female, age 77, widowed) Another participant explained, “*We could talk about you know what was happening in our lives at that point […] Marking milestones, so people knew if there was an anniversary date coming up or if there was something that was potentially going to be a challenge, there was that follow up when we would come back together and people would say “How did it go?” and people understood. They got it.*” (female, age 67, widowed) This underscored how certain times of the year hold unique significance for individuals, making it especially important to be able to discuss and receive support during those moments to help them navigate emotionally challenging yet meaningful days.

The open-group also enabled participants to balance their needs alongside the shifting emotions that arose during their grieving process, as stated by a participant, “*You talk or you don’t talk. You know, you have a good day or you have a bad day.*” (female, age 73, widowed) Additionally, participants naturally connected with each other on their own terms, guided by what they felt was most important to them: “*Because there was no formal program per se, it took on a life of its own. People talked about what was current, what was important at that time. And I think that there is a huge value in that.*” (female, age 67, widowed) The informal conversations about grief during these sessions were particularly valued by many, where one participant stated, “*We talked about, you know, our daily lives. We talked about everything that was going on, where we’re at. We got suggestions from each other, how we were managing with our days, what to do to keep ourselves busy. So it was nice to share all that.*” (female, age 68, widowed)

Whereas the unstructured agenda in the open-group was perceived positively, a few acknowledged differing preferences. A participant noted: “*To offer a program like that for the people that it fits with is important. If Hope & Cope is offering some type of grieving opportunities for people, then you have to be diverse.*” (female, age 74, widowed)

**Structured focus on grief.** In the closed-group, each session had set objectives and topics. These are summarized in Supplementary Materials Figure S1. Most participants reflected on how these validated their emotions, gave them the sense that the program was thoughtfully structured, and helped them gain a deeper understanding of their own grieving experiences. The perceived benefits of structured support were further explained by one participant, “*Don’t forget when there’s you, your partner, you become in a relationship very structured. […] We all are creatures of habit […] But once you’re standing on your own, instead of standing with that person, some of that structure falls apart. Some of that structure falls to the wayside.*” (male, age 77, widowed)

Moreover, structured discussions that focused on the experiences of loss and grief encouraged participants to share more freely. One participant stated, “*If we just talked about other things besides grief, then it wouldn’t have been so easy to bring this up but because we’re talking about grief and how we deal with it and our feelings of about loss, it made it a lot easier for me to be open to speaking about my experiences and my feelings.*” (female, age 62, lost parent)

Although participants generally responded positively to the topics covered in the *Living with Loss* (closed-group) program, two felt some of the content was not personally relevant. One reported, “*At one point, there was something like denial. I said, ‘How can I deny the deaths?’ I didn’t deny it, ever. Anger, I didn’t feel either. Why would I feel anger? It’s not his fault in dying.*” (female, age 83, lost child)

Closed-group participants also pointed out that the program’s structured content precluded opportunities for informal conversations that could enhance people’s experiences. Two participants reported:

“*There were times where because it’s so structured, they have to get everything in and the session is only an hour and a half. That’s why maybe it’s better to have let’s say 9 or 10 sessions and doing your structure, but not so much in one session so that you allow the people to talk about it and understand it.*”

(female, age 65, widowed)

“*Any activities in which you can have more informal discussions. You could also have small groups, for instance.*”

(female, age 83, widowed)

#### 3.2.3. Theme 3. Grief Support Dynamics in Relation to Group Composition

**Benefits and challenges of participant turnover.** The flexibility to freely join the open-group program was positively received by participants: “*It was very easy. I was welcomed.*” (female, age 67, widowed) Such flexibility was valued based on the challenges they previously faced while waiting to join a closed-group support program. One participant shared, “*it’s kind of hard to know that there is a program out there that I was interested in that I was refused to attend. I couldn’t understand the reason. I mean. I thought support was supposed to be support, you know? That’s what I thought. I was very disappointed.*” (female, age 68, widowed) This challenge was also recognized by closed-group participants as a fixed start date meant that new members could not join once the program had begun. One participant asked, “*what happens to people that have lost loved ones in the interim [of Living with Loss]?*” (female, age 64, widowed)

According to open-group participants, the flexibility to join at any time had limitations. They explained, “*whenever there were new people joining the group, [facilitator] would introduce them and they would say why they were there. We’d also say our name and why we were there, and that was a little difficult at times.*” (female, age 74, widowed) Similarly, in a closed-group, participants noted that the first session was the most challenging and uncomfortable as it was their first time meeting one another. That said, within the closed-group, as “*most of the participants were there on a regular basis,*” (female, age 73, widowed), the consistent presence of the same individuals promoted stability and interpersonal connections, which contributed to fostering a strong sense of community. Such comments included:

“*Because we met every week or every two weeks, it was a little bit easier to speak because these are people that I’ve seen.*”

(female, age 62, lost parent)

“*I would have to say that the last sixth, seventh and eighth [sessions], I think it has everything to do with the fact that we feel comfortable. You know each other’s names and you know more about their loved ones. You share more and in sharing more, that brings comfort […]. It becomes a family.*”

(female, age 64, widowed)

**Shared and diverse grief stages.** Interacting with individuals at similar stages of grief within the closed-group setting was found to be beneficial, allowing most (8/11) to share their own experiences more freely while feeling less alone in their journey. Two participants stated:

“*Because of the fact that these individuals were experiencing what I was experiencing, it allowed me to open up and to share even more. Things that I wouldn’t necessarily share.*”

(female, age 64, widowed)

“*I think it helps to see that you’re not alone in this situation.*”

(female, age 83, widowed)

A participant was also keen to connect with individuals at different stages of grief, recognizing the value in diverse perspectives. She underscored that, “*even if it’s ten years ago that somebody lost a child, initially that person went through the same things. I would be open to what that person did in their life to kind of accept it, and say, ‘Well, if she did it, maybe I can do it too,’ you know?*” (female, age 83, lost child)

*Mourning Walk* was open to any individual who had lost a loved one to cancer at any point in time. The value of connecting with individuals at different stages of grief was indeed highlighted by participants (4/7). They noted that it allowed for the exchange of helpful coping strategies and offered a sense of hope. Two participants explained:

“*We had people at different stages of grief. You know, some had just lost their spouse recently. Others have been there for three or four years. […] you see the stepping stones when you can speak to people, how they managed, how they’re moving on, what they’re looking forward to. So it helps you to realize that, yeah, that there’s a chance for me too, that I’m going to get through it.*”

(female, age 68, widowed)

“*I think what helped me was seeing the steps people made. For instance, people traveling to places they’ve been, where they’d always go in with their loved one and going by themselves. It was sort of like being in this panopticon of being able to see all the possible futures at a point and then just really cheering for that person […] And that sort of the cheering someone on is kind of a self-fulfilling gift because it helps people who are being cheered, but it also really helps the people who are doing the cheering because it brings these things into the realm of the possible for the people who are not there yet.*”

(female, age unknown, lost parent)

Moreover, an open-group set up offered benefits not only to individuals who had recently experienced a loss but also to those who had been bereaved for longer. A participant reported, “*as someone who’s been there for a while now, I can look back and see, you know, a year ago, I couldn’t talk about this. Two years ago, I certainly couldn’t talk about this. I guess I have come a long way. So, I think that there’s, you know, there are benefits for whatever stage you are in that process.*” (female, age 67, widowed), thus recognizing their personal growth through shared dialogue.

## 4. Discussion

This study provides a unique perspective on cancer bereavement support programs by exploring user experiences with open- and closed-group formats. Participants highlighted the subjective and individual nature of bereavement, suggesting that support interventions should be tailored to align with the unique needs and preferences of those seeking support. Whereas a given bereavement program may not meet the needs of all individuals, our findings suggest that timely, consistent, and ongoing support—paired with the flexibility to balance personal needs with program participation—was broadly valued by bereaved individuals.

A significant finding was the importance of ongoing support. Unresolved grief and spousal loss are predictive factors for complicated or long-term grief [15,40]. Moreover, grief disorders are prevalent in approximately 10–20% of cancer-bereaved families [15,16]. According to the participants, grief has a lasting nature. As one’s grief experience can depend on a variety of factors such as the disease type, sociodemographic characteristics, and psychosocial factors [41,42,43], grief processes do not have set timelines. This may explain why some closed-group participants expressed a desire for additional sessions at program completion to continue their healing process. While prior qualitative research suggests bereavement support groups (often studied in closed-group settings) can be beneficial in terms of offering a sense of relief, normalizing grief experiences and reducing social isolation [44,45,46], quantitative literature presents mixed findings regarding their effectiveness in bereavement support. For example, a study investigating a five-week bereavement group intervention reported unchanged levels in participants’ levels of grief, depression, or anxiety post-intervention compared to the control group. At one-year follow-up, grief levels of those who received the intervention remained unchanged [47]. Conversely, Jerome et al. [23] observed significant reductions in participants’ grief and symptoms of depression and anxiety after attending a six-session cancer bereavement group, and a maintenance of reduced levels of grief and depression at 3 months. According to a meta-analysis, significantly lower grief and depressive symptoms reported at post-intervention can become non-significant over time [48]. Overall, evidence of the long-term effectiveness of bereavement support groups remains uncertain. Together, these findings suggest that while closed bereavement support groups can provide meaningful benefits, the limited number of sessions may not be optimal for some. Adapting the program structure and length according to needs may enhance the program’s impact.

Given that grief is a universal response to loss, several theories have been proposed to explain patterns of grief processing. A well-known framework commonly applied to grief is the “Five stages of dying”, identifying the common patterns to grief reactions including denial, anger, and depression [49]. Indeed, many participants in the closed-group found grief-related topics discussed in the sessions- such as denial and anger- to be both beneficial and relevant. However, some pointed out that program topics were not always relevant to their experiences. These further highlight individual variations in processing grief, necessitating a more nuanced and personalized approach [50,51]. Given the perceived benefits and limitations of both open and closed group formats identified, engaging in in-depth discussions with bereaved individuals prior to participation may be a valuable step in ensuring the support received best aligns with their needs.

The findings also underscore the importance of timely access to bereavement support. This is particularly relevant given the evidence that bereaved family members often experience high levels of distress within the first two years following their loss [52]. Furthermore, research indicates that the quality of care provided to both patients and informal caregivers is significantly associated with bereavement outcomes [53,54,55]. This highlights the critical role of the health care team, including trained volunteers—particularly in palliative care and end-of-life care—in identifying and supporting family members in need [56]. Nurses, in particular, interact with patients and families regularly, developing trust and anticipating their needs [56,57,58]. Maintaining a presence across the cancer care trajectory can help them prepare for loss and adjust their grief experiences [59]. This allows nurses to identify, discuss, and prioritize families’ beliefs and values which can enable timely and appropriate support [11]. Unfortunately, in many settings, follow-up after a patient’s death is not routinely performed, leaving significant gaps in bereavement support [60,61]. Enhancing collaboration among healthcare providers and community-based organizations can improve access to and outcomes related to bereavement support.

### 4.1. Study Limitations

This study has inherent limitations. First, because participants voluntarily agreed to take part in the study, self-selection bias may have been operative. Second, although open- and closed-group comparisons are valuable, program attendance was quite variable within open-group participants when compared to the closed-group. Last, representativity is limited as participants mainly identified as white, well-educated, and female.

### 4.2. Future Directions

Our findings revealed that program format—such as open versus closed-group—can shape participant experiences, bereavement-related processes, and outcomes. Future research should explore additional features of bereavement programs, such as delivery mode (e.g., virtual, in-person), attendees’ background characteristics, and group facilitator experience and style. Herein, open-group participants reported enhanced propensity to identify and attend to their grief-related needs as these emerged when compared to closed-group participants. Longitudinal mixed-method studies could help document how program benefits may evolve and be sustained over time. In addition, the study sample consisted exclusively of individuals living alone. Further research is warranted to document the support needs of bereaved individuals who live with others or have dependents. Last, controlled trials should systematically test the extent to which personalized programs affect program attendance and bereavement outcomes.

## 5. Conclusions

This qualitative study begins investigating participant perspectives regarding program delivery formats. The findings revealed that open versus closed formats confer distinct strengths and limitations related to participant experiences. Findings indicate that open-groups facilitated personalized and on-demand grief support, whereas closed- groups offered structure in format and content, as well as shared experiences as grief progressed. Some drawbacks included participant turnover in open-groups and, in closed-groups, a firm program end date. In both groups, participants valued support that was timely, consistent, and ongoing, along with the flexibility to balance their personal needs with program participation requirements. The findings underscore the importance of considering multiple features of supportive bereavement programs in their design, content, delivery, and expected impact. Given the unique experiences of bereaved individuals, supportive interventions should continue to favor tailoring according to people’s evolving needs and preferences.

## Figures and Tables

**Table 1:** Semi-structured interview guide domains and related questions.

Domains	Post-Program Interview Questions
(1) Affective attitude	Is there anything you liked about (the program)?
(2) Burden	How easy or difficult was it to participate in (the program)?
(3) Effectiveness	To what extent do you think (the program) made a difference in the lives of people attending it?
(4) Opportunity costs	Is there anything in particular that you personally had to give up or sacrifice to participate in (the program)?
(5) Intervention coherence	Do you have a clear understanding of the objectives of the program you were enrolled in?
(6) Self-efficacy	In your opinion, how confident are you that you were able to perform the tasks required by (the program)?
(7) Ethicality	To what extent do you think (the program) was a good fit with your own values or beliefs?

**Table 2:** Participant characteristics (*N* = 18).

Participant Characteristics	*N* = 18 (Total Sample)	% (Total Sample)	*n* = 11 (Living with Loss)	*n* = 8 (Mourning Walk)
**Biological sex**				
Female	16	88.9	9	7
Male	2	11.1	2	0
Age (years) *				
30–39	1	5.6	1	0
40–49	0	0	0	0
50–59	1	5.6	0	1
60–69	5	27.8	3	2
70–79	7	38.9	4	3
80–89	3	16.7	3	0
**Marital status**				
Widowed	15	83.3	9	6
Separated/divorced	1	5.6	0	1
Single (or never married)	2	11.1	2	0
**Ethnicity**				
White (Canadian/European)	15	83.3	9	6
Latin American	2	11.1	1	1
Southeast Asian	1	5.6	1	0
**Currently Living with Someone**				
Yes	0	0	0	0
No	18	100	11	7
**Dependents**				
None	18	100	11	7
**Level of Education completed**				
Undergraduate	6	33.3	4	2
Graduate	5	27.8	2	3
High school	3	16.7	3	0
Technical or vocational school or pre-university degree	4	22.2	2	2
**Work status**				
Retired	12	66.6	7	5
Full-time (>30 h/week)	1	5.6	1	0
Part-time (<30 h/week)	1	5.6	0	1
Self-employed	2	11.1	2	0
Disability/sick leave	2	11.1	1	1
**Relationship to Deceased**				
Spouse	14	77.8	8	6
Parent	1	5.6	1	0
Child	3	16.6	2	1

* Adds up to less than 100% due to non-responses.

**Table 3:** Main themes and subthemes.

Main Themes	Subthemes
1. Program structure according to grief timeline	1.1 Ongoing access to support (open) 1.2 Perceived progress (open) 1.3 Consistent support (open and closed)
2. Flexibility in the choice of topics and impact on grief experiences	2.1 Balancing dynamic and timely needs (open) 2.2 Structured focus on grief (closed)
3. Grief support dynamics in relation to group composition	3.1 Benefits and challenges of participant turnover (open) 3.2 Shared (open) and diverse (closed) grief stages

## Data Availability

Data from this study are available from the authors upon request.

## Supplementary Materials

The following supporting information can be downloaded at: https://www.mdpi.com/article/10.3390/curroncol32090505/s1, Figure S1: Hope & Cope’s Living with Loss program topics; Figure S2: Assessment of the qualitative study using the COREQ checklist.
