# Supplementary material for: Participants’ Perceptions of Advantages and Drawbacks of “Drop-In” Versus “Closed-Group” Formats Related to Cancer Bereavement Program Delivery
Source: Curr Oncol. 2025 Sep 10;32(9):505. doi: 10.3390/curroncol32090505 (PMC12468342; doi:10.3390/curroncol32090505)
Supplement: Supplementary file 1 [file curroncol-32-00505-s001.zip › Figure S1. Hope & Cope's Living with Loss program topics.pdf]

Article: **Participants' Perceptions of Advantages and Drawbacks of "Drop-in" versus "Closed-group" Formats Related to Cancer Bereavement Program Delivery**

Supplementary Material

Figure S1. *Hope & Cope*'s *Living with Loss* program topics

Living with Loss	**Topics**
Session 1	Welcome – Guidelines for the group – sharing stories
Session 2	Symptoms of grief and typical reactions
Session 3	Feelings and emotions associated with grief
Session 4	Coping and support
Session 5	Spirituality and significant days
Session 6	Memories: Sharing photos and keepsakes
Session 7	Grief and music
Session 8	Finding Meaning, connection, acceptance – Introspection/Where do I go now?
