# Supplementary material for: Participants’ Perceptions of Advantages and Drawbacks of “Drop-In” Versus “Closed-Group” Formats Related to Cancer Bereavement Program Delivery
Source: Curr Oncol. 2025 Sep 10;32(9):505. doi: 10.3390/curroncol32090505 (PMC12468342; doi:10.3390/curroncol32090505)
Supplement: Supplementary file 1 [file curroncol-32-00505-s001.zip › Figure S2. Assessment of the qualitative study using the COREQ checklist.pdf]

Article: **Participants' Perceptions of Advantages and Drawbacks of "Drop-in" versus "Closed-group" Formats Related to Cancer Bereavement Program Delivery**

Supplemental Material

Figure S2. Assessment of the qualitative study using the COREQ [1] checklist

**Domain**		**Item**	**Description**
**1. Research team and reflexivity**	*Personal characteristics*	Interviewer	First and senior authors
		Credentials	B.Sc. (first author) and PhD, FCAHS, RN (senior author)
		Occupation	Graduate student (first author) and Full Professor (senior author)
		Gender	Female (first and senior authors)
		Experience and training	Coursework (first author) and 25-year research and mentoring experience (senior author)
	Relationship with participants	Relationship established	Initial relationship was established via phone screening by first author (p. 3)
		Participant knowledge of the interviewer	Participants were informed of the purpose of the study and reasons for research (p. 3)
		Interviewer characteristics	Reasons for the research topic was shared with participants during phone screening (p. 3)
**2. Study design**	*Theoretical framework*	Methodological orientation and theory	Reflexive thematic analysis using inductive approach (p. 4)
	Participant recruitment	Sampling	Convenience sampling (p. 3)
		Method of approach	Participants recruited by phone (p. 3)
		Sample size	N = 18 (p. 3+)

		Non-participation	n = 6 (p. 3)
	Setting	Setting of data collection	Virtual (Zoom conferencing platform), in-person (Jewish General Hospital, Montreal, Quebec, Canada) and by phone (p. 3)
		Presence of non-participants	No one except participants and researchers (p. 4)
		Description of sample	Predominantly female (16 females, 2 males), age ranged between 31 and 89 years, 15 participants identified as Caucasian (p. 5)
	Data collection	Interview guide	Semi-structured interview using Sekhon et al. [2] guide on intervention acceptability (p. 4)
		Repeat interviews	No
		Audio/visual recording	Interviews were audio-recorded (p. 4)
		Field notes	Field notes were recorded during interviews
		Duration	Interviews lasted between 30 and 60 minutes (p. 4)
		Data saturation	Saturation was reached and discussed (p. 4)
		Transcripts returned	Transcripts were not returned to participants
**3. Analysis and findings**	*Data analysis*	Number of data coders	2 researchers coded the data (p. 4)
		Description of coding tree	A table of main themes and subthemes provided (p. 6)

		Derivation of themes	Themes were derived from the data using an inductive approach (p. 4)
		Software	Microsoft Word and Excel (p. 4)
		Participant checking	Participants did not provide feedback on the findings
	Reporting	Quotations presented	Verbatim quotes presented under each theme/sub-theme with each quote identified by participant sex, age and relationship to deceased (pp. 6-10)
		Data and findings consistent	There is consistency between data presented and findings
		Clarity of major themes	Major themes are outlined and presented in results (pp. 6-10)
		Clarity of minor themes	Minor themes are discussed (pp. 6-10)

References

1. Tong, A.; Sainsbury, P.; Craig, J. Consolidated Criteria for Reporting Qualitative Research (COREQ): A 32-Item Checklist for Interviews and Focus Groups. *Int J Qual Health Care* **2007**, *19* (6), 349–357. https://doi.org/10.1093/intqhc/mzm042.
2. Sekhon, M.; Cartwright, M.; Francis, J. J. Acceptability of Healthcare Interventions: An Overview of Reviews and Development of a Theoretical Framework. *BMC Health Services Research* **2017**, *17* (1), 88. https://doi.org/10.1186/s12913-017-2031-8.
